# Comparing subjective quality of recovery between remimazolam- and propofol-based total intravenous anesthesia for surgical procedures: a meta-analysis

**DOI:** 10.1186/s13643-024-02660-8

**Published:** 2024-09-17

**Authors:** Kuo-Chuan Hung, Wei-Ting Wang, Wei-Cheng Liu, Chih-Wei Hsu, Yen-Ta Huang, Jheng-Yan Wu, I-Wen Chen

**Affiliations:** 1 Department of Anesthesiology, Chi Mei Medical Center, Tainan City, Taiwan; 2 Department of Anesthesiology, E-Da Hospital, I-Shou University, Kaohsiung City, Taiwan; 3 The Department of Occupational Therapy, Shu-Zen junior College of Medicine and Management, Kaohsiung City, Taiwan; 4 Department of Psychiatry, Kaohsiung Chang Gung Memorial Hospital and Chang Gung University College of Medicine, Kaohsiung City, Taiwan; 5 Department of Surgery, National Cheng Kung University Hospital, College of Medicine, National Cheng Kung University, Tainan City, Taiwan; 6 Department of Nutrition, Chi Mei Medical Center, Tainan City, Taiwan; 7 Department of Anesthesiology, Chi Mei Medical Center, No.201, Taikang Taikang Vil., Liouying Dist., Liouying, Tainan City, 73657 Taiwan

**Keywords:** Remimazolam, Propofol, Quality of recovery, QoR, General anesthesia

## Abstract

**Background:**

Remimazolam is a novel ultra-short-acting benzodiazepine that has been recently introduced as an alternative to propofol for general anesthesia. While both agents have been compared in terms of safety and efficacy, their relative effects on postoperative quality of recovery (QoR) remain unclear. Therefore, this meta-analysis aimed to compare the effects of remimazolam and propofol on subjective QoR in surgical patients who underwent general anesthesia.

**Methods:**

Medline, Embase, Google Scholar, and the Cochrane Central Register of Controlled Trials were searched from inception to May 28, 2024 to identify randomized controlled trials comparing remimazolam and propofol in terms of postoperative QoR. The Cochrane risk-of-bias tool (RoB 2) was used to assess study quality. QoR score on postoperative day (POD) 1 (primary outcome), QoR scores on PODs 2–3, QoR dimensions, time to loss of consciousness, other recovery characteristics, and rescue analgesia requirement were evaluated using random-effects meta-analyses.

**Results:**

This meta-analysis included 13 studies published between 2022 and 2024 involving 1,418 patients. QoR was evaluated using either the QoR-15 (10 studies) or QoR-40 (3 studies) questionnaire. The pooled results indicated no significant difference in the QoR scores on POD 1 (standardized mean difference: 0.02, 95% confidence interval [CI]: − 0.20, 0.23, *P* = 0.88, I^2^ = 73%) and PODs 2–3 between remimazolam and propofol. Furthermore, no significant differences were observed in QoR dimensions, length of postanesthesia care unit (PACU) stay, and time to extubation as well as in the risks of agitation and postoperative nausea and vomiting. Patients administered remimazolam exhibited slower anesthetic induction (mean difference (MD): 32.27 s) but faster recovery of consciousness (MD: − 1.60 min) than those administered propofol. Moreover, remimazolam was associated with a lower risk of rescue analgesia requirement in the PACU (risk ratio: 0.62, 95% CI: 0.43, 0.89, *P* = 0.009, *I*^*2*^ = 0%) but not in the ward.

**Conclusion:**

Remimazolam is a potential alternative to propofol for general anesthesia as it offers similar QoR to the latter and has advantages in terms of consciousness recovery and immediate postoperative analgesia requirement.

**Supplementary Information:**

The online version contains supplementary material available at 10.1186/s13643-024-02660-8.

## Introduction

Postoperative subjective quality of recovery (QoR) refers to the patient’s own perspective on how they feel during the postoperative period, which can significantly differ from the clinical or objective measures of recovery [1, 2]. Several tools and questionnaires have been developed to systematically evaluate subjective QoR, including QoR-40 [3, 4] and its shorter version, QoR-15 [5]. These instruments evaluate multiple domains of recovery (e.g., physical comfort and emotional well-being) to provide a comprehensive overview of the patient’s subjective recovery experience. In patients undergoing elective surgeries, poor QoR has been associated with postoperative complications up to 30 days [6]. Furthermore, research has shown that patients with suboptimal recovery quality were less likely to achieve disability-free survival three months after abdominal surgery [7], highlighting the importance of enhancing subjective QoR in clinical settings. In recent years, improvement of patient care by using prophylactic agents (e.g., lidocaine) or different anesthetic techniques to enhance subjective QoR has gained attention [8–14].

Propofol and volatile anesthetic agents are commonly used in general anesthesia, enabling patients to undergo invasive treatments without experiencing pain or awareness. Compared with volatile agents, propofol-based anesthesia may offer favorable recovery profiles, such as better subjective QoR, reduced postoperative pain, and lower incidence of postoperative nausea and vomiting (PONV), emergence agitation, and postoperative cognitive dysfunction [15–17]. Remimazolam is a novel ultra-short-acting benzodiazepine that acts on gamma-aminobutyric acid (GABA) receptors to induce sedation, hypnosis, and amnesia [18–20]. Compared with other benzodiazepines, such as midazolam, remimazolam has a more rapid onset and offset of action owing to its unique pharmacokinetic profile [18–20]. It undergoes rapid hydrolysis by tissue esterases, resulting in a shorter duration of action and a more predictable recovery profile [18–20]. Compared with propofol, emerging evidence indicates that remimazolam is associated with lower risks of hypotension, hypoxemia, and other adverse events (e.g., bradycardia and pain at the injection site) [21–26]. These characteristics make remimazolam an attractive choice for procedural sedation and general anesthesia as it enables quicker patient recovery and improved cardiopulmonary stability. However, studies comparing subjective QoR between propofol and remimazolam are scarce. To the best of our knowledge, only two recent meta-analyses [24, 27] have evaluated the subjective QoR between these two agents as a secondary outcome. However, these meta-analyses had notable limitations. First, each meta-analysis included only two studies, leading to insufficient statistical power to identify potential differences. Second, they [24, 27] did not examine the possible impact of key factors such as the duration of surgery or the dosage of remimazolam on the observed effect size.

To enable a comprehensive and reliable assessment of the comparative effects of remimazolam and propofol on postoperative QoR, a meta-analysis of randomized controlled trials (RCTs) involving surgical patients who underwent general anesthesia was conducted to increase statistical power and resolve the knowledge gap in the existing literature.

## Methods

This systematic review was registered on PROSPERO (CRD42024551877). The reporting of this meta-analysis adhered to the guidelines set forth by the Preferred Reporting Items for Systematic Reviews and Meta-Analyses (PRISMA).

### Search strategies

Medline, Embase, Google Scholar, and the Cochrane Central Register of Controlled Trials (CENTRAL) were searched from their inception until May 28, 2024 to identify RCTs comparing the effects of remimazolam and propofol on postoperative QoR in patients who underwent general anesthesia for any type of surgery. The search strategy included the use of a combination of keywords and MeSH terms pertaining to remimazolam, propofol, surgery, and postoperative recovery. Specific search strings for one of the databases are presented in Supplemental Table 1. To capture all relevant studies, the reference lists of the selected studies and published meta-analyses comparing the differences between remimazolam and propofol were also manually reviewed. The search included all studies on humans, and no restrictions were imposed on publication date or language. Two reviewers independently evaluated the titles and abstracts of the retrieved records based on predefined inclusion criteria, followed by a full-text assessment of potentially eligible studies by the same reviewers. Any disagreements were resolved through discussion or by involving a third reviewer.

### Eligibility criteria

The inclusion criteria were as follows: (1) studies involving adult patients (i.e., age ≥ 18 years) who underwent general anesthesia for any type of surgery, (2) RCTs using remimazolam as the intervention and propofol as the control for maintaining general anesthesia, and (3) studies reporting at least one subjective outcome measure related to postoperative QoR, such as QoR-15 or QoR-40.

The exclusion criteria were as follows: (1) nonrandomized or observational studies; (2) studies involving pediatric patients, sedative procedures (e.g., colonoscopy), or regional anesthesia techniques; (3) studies not reporting any relevant outcome measures related to postoperative QoR; and (4) studies with duplicate publications or containing insufficient data for meta-analysis.

### Outcome definition

The primary outcome of this meta-analysis was the QoR score on postoperative day (POD) 1. QoR score is a validated, patient-reported outcome measure that evaluates the quality of postoperative recovery across multiple domains, including physical comfort, emotional state, physical independence, psychological support, and pain. Higher QoR scores indicate better postoperative recovery. For studies reporting QoR scores using different scales (e.g., QoR-15, QoR-40), the standardized mean difference (SMD) was calculated to facilitate comparison across studies. The SMD expresses the size of the intervention effect in each study relative to the variability observed in that study, allowing for the comparison and pooling of results from studies that used different QoR scales.

The secondary outcomes included QoR scores on PODs 2–3, QoR dimensions (e.g., physical comfort, emotional state, physical independence, psychological support, and pain), time to loss of consciousness (LOC), time to recovery of consciousness (ROC), time to extubation, emergence agitation, length of postanesthesia care unit (PACU) stay, PONV, and rescue analgesia requirement in the PACU or ward.

### Data collection

Data extraction was independently conducted by two reviewers using a standardized form. Detailed information was collected from each study, including study characteristics (i.e., first author, publication year, design, sample size, and country), participant demographics (i.e., age, sex, body mass index [BMI], physical status, surgical type, surgical duration), and specifics of interventions and comparisons (i.e., dosing and administration of remimazolam and propofol). Outcomes such as QoR scores for PODs 1 and 2–3, other QoR dimensions, time to LOC, time to ROC, time to extubation, emergence agitation, length of PACU stay, PONV, and rescue analgesia requirement in the PACU or ward were also recorded. Discrepancies between the reviewers were resolved by consensus, and study authors were contacted for any missing or unclear data. If the data remained unattainable, studies were excluded from specific analyses.

### Risk-of-bias assessment

The risk of bias in our analyzed studies was independently evaluated by two reviewers using the Cochrane risk-of-bias tool (RoB 2) for RCTs. This tool assesses five domains: bias from the randomization process, bias due to deviations from intended interventions, bias from missing outcome data, bias in outcome measurement, and bias in the selection of reported results. The risk of each domain was classified as low, some concerns, or high. Subsequently, the overall risk for each study was determined based on these assessments. Disagreements between reviewers were resolved through discussion or consultation with a third reviewer if necessary.

### Certainty of evidence

The certainty of evidence for each outcome was evaluated using the Grading of Recommendations, Assessment, Development, and Evaluation (GRADE) approach. GRADE categorizes evidence into four levels: high, moderate, low, and very low. The outcomes from the meta-analysis of RCTs initially have a high certainty rating. But such a high rating may be downgraded due to risk of bias, inconsistency, indirectness, imprecision, and publication bias. Two reviewers independently evaluated the certainty of each outcome using the GRADE approach. Disagreements were resolved through discussion or consultation with a third reviewer if necessary.

### Statistical analysis

The statistical analysis for our meta-analysis was conducted using Cochrane Review Manager (RevMan, version 5.4). The SMD or mean difference (MD) with 95% confidence intervals (CIs) for continuous outcomes and the risk ratio (RR) with 95% CIs for categorical outcomes were calculated. For studies that reported continuous outcomes as medians with interquartile ranges (IQRs), we converted these values to means and standard deviations (SDs) to facilitate the meta-analysis. The conversion was performed using the method described by Wan et al. [28]. A random-effects model was used in all analyses to address variability both within and between studies. To avoid overlapping sample sizes in studies with multiple intervention groups, a strategy where participants in the control groups were divided into subgroups was employed. Each subgroup was then compared with its corresponding specific intervention arm using a previously described method [29]. *P*-value < 0.05 was considered to indicate statistical significance.

Heterogeneity was evaluated using the *I*^*2*^ statistic, considering an *I*^*2*^ value above 75% as indicative of substantial heterogeneity. In cases of significant heterogeneity in the primary outcome (i.e., QoR scores on POD 1), subgroup analyses were conducted to identify potential sources based on the surgical duration (i.e., < 60 min vs. > 60 min) or the type of outcome measurement (i.e., QoR-15 and QoR-40). Publication bias was assessed using funnel plots when more than 10 studies or datasets were available for a given outcome. Sensitivity analyses were conducted to evaluate the robustness of the meta-analysis results. First, we sequentially excluded individual studies to assess the influence of each study on the pooled effect estimates. Second, we re-ran the analyses after removing studies deemed to have a high risk of bias to evaluate the impact of study quality on the results.

Meta-regression analyses were conducted to explore the potential influence of the duration of surgery or remimazolam dose on the primary outcome (i.e., QoR scores on POD 1). Remimazolam dosage or surgical time were treated as continuous variables. In cases where the dosage was reported as a range, the midpoint of the range was used. Studies that did not report remimazolam dose or surgery duration were excluded from the meta-regression analysis. Analyses were conducted using Comprehensive Meta-Analysis (Version 4, Biostat, Englewood, NJ, USA). In addition, trial sequential analysis (TSA) was conducted to verify the adequacy of the information size and the robustness of our primary outcome. The TSA focused on the QoR-15 scale, which is the most frequently used measure. According to previous research, the minimum clinically important difference for the QoR-15 scale is 6.0 [30]. Consequently, the TSA settings included an alpha level of 0.05, a power of 80%, and a MD of 6.

## Results

### Study selection

**Fig. 1:**
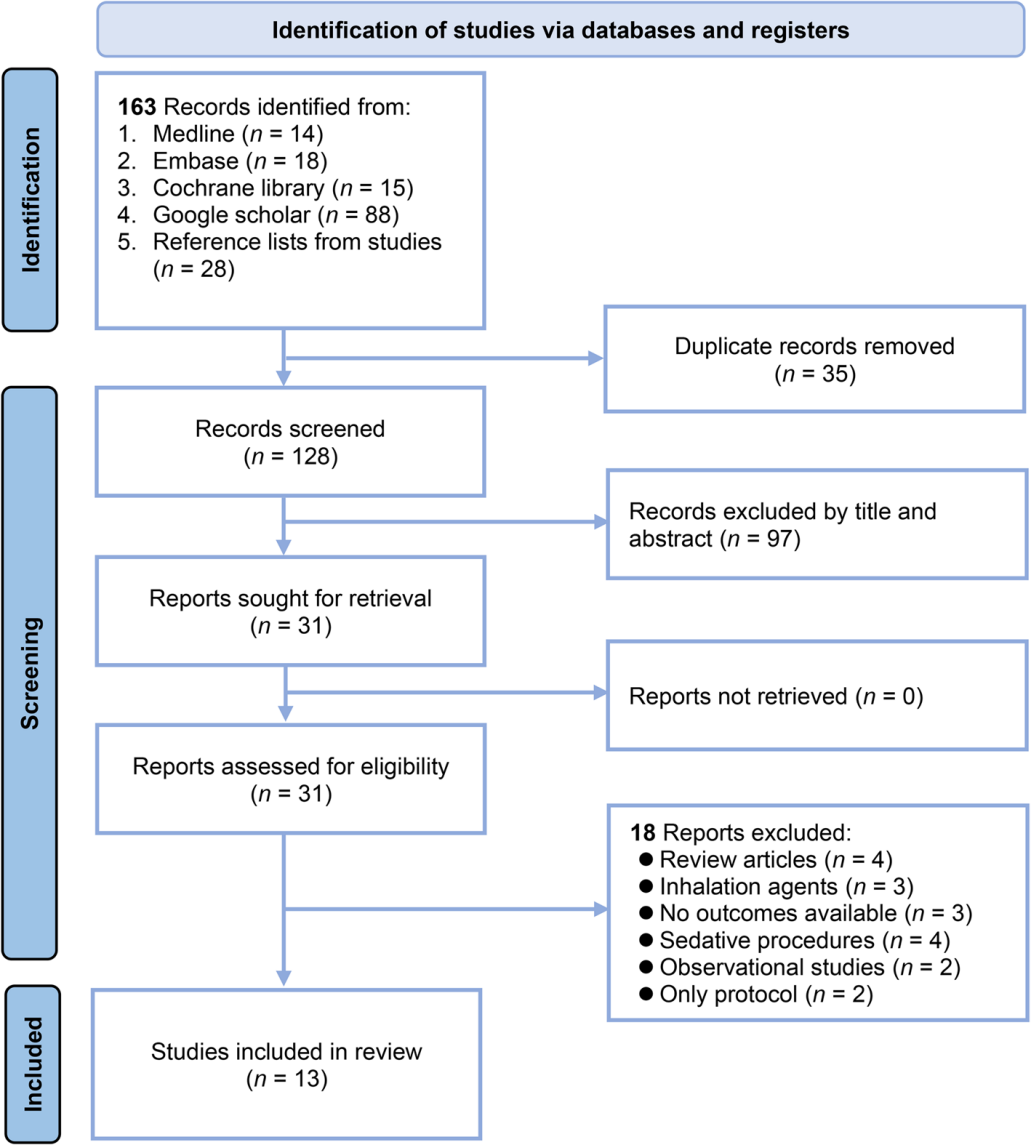
Flowchart for studies selection

The systematic literature search yielded 163 records from four databases and reference lists from three relevant meta-analyses [24, 27, 31] (Fig. 1). After eliminating 35 duplicates, 128 records were screened by title and abstract, excluding 97 that did not meet the inclusion criteria. The full texts of the remaining 31 reports were assessed for eligibility, which led to the exclusion of additional 18 studies for the following reasons: review articles (*n* = 4), studies involving inhalation agents (*n* = 3), lack of available outcomes (*n* = 3), studies focusing on sedative procedures (*n* = 4), observational studies (*n* = 2), and articles presenting only the study protocol (*n* = 2). Consequently, 13 RCTs published between 2022 and 2024 involving 1418 patients were included in the systematic review and meta-analysis [32–44]. Two [34, 43] of the thirteen studies were not retrievable from major databases (i.e., Medline or Embase) and were primarily sourced from Google Scholar. Additionally, although one study [42] was listed in Embase, it is available only in Chinese.

### Characteristics of the studies

The main characteristics of the 13 RCTs included in this meta-analysis are summarized in Table 1. These studies were conducted in China (*n* = 7), Korea (*n* = 5), and Japan (*n* = 1) and involved a total of 1,418 participants. The sample sizes of the individual studies ranged from 36 to 192 patients. The mean age of the participants ranged from 39 to 85 years, and the proportion of male patients varied from 0 to 70%. Majority of the studies included patients with American Society of Anesthesiologists (ASA) physical status I–II, whereas four [33, 36, 38, 40] included patients with ASA III. The surgical procedures performed in the studies were diverse, with the mean surgical duration ranging from 9 to 286 min. The intraoperative infusion dose of remimazolam ranged from 0.3 to 3 mg/kg/h, whereas the propofol dose ranged from 2 to 10 mg/kg/h or was administered as a target-controlled infusion with effect-site concentrations ranging from 1 to 6 μg/mL. Remifentanil was the most commonly utilized opioid during surgery. The anesthetic depth was monitored using the bispectral index in most studies, with a target range of 40–60. Two studies [36, 38] used the patient state index to monitor the anesthetic depth, with a target range of 25–50. The QoR was evaluated using either the QoR-15 or the QoR-40 questionnaire. QoR-15 was used in 10 studies [32, 34, 36–38, 40–44], whereas QoR-40 was used in the remaining 3 studies [33, 35, 39].

**Table 1:** Characteristics of 13 randomized controlled trials with a total of 1418 participants

Studies	Mean age (years)^c^	Male (%)^c^	BMI (kg/m^2^)^c^	N	ASA	Procedure	Surgical Time (min)^c^	Dosage (R) mg/kg/h^b^	Dosage (P) mg/kg/h^b^	Opioid^b^	BIS	QoR	Country
Chen 2024 [43]	72/72	47/51	25/24	110	I-II	Fundus surgery	97/98	1–2	4–10	remi	40–60	QoR-15	China
Choi 2022 [32]	40/41^d^	0	NA	139	I-II	Thyroidectomy	53/59	1–2	2–6 ug/ml^f^	remi	na	QoR-15	Korea
Huang 2023 [33]	63/64	0	24/25	120	II-III	Breast surgery	98/98	0.3	2	remi	40–60	QoR-40	China
Jiao 2024 [34]	41/40	56/60	24/24	90	I-II	VCP	9/9	0.5–1	4–6	NA	40–60	QoR-15	China
Kim 2023 [35]	42/43	65/62	24/24	189	I-II	OMS	43/46	1–2	3–5 ug/mL^f^	remi	40–60	QoR-40	Korea
Kotani 2024 [36]	83/85	35/33	NA	36	III	TAVI	NA	NA	2.5 ug/mL^f^	remi	25–50^e^	QoR-15	Japan
Lee 2023 [37]	45/51	25/34	24/23	57	I-II	Thyroidectomy	85/85	2	2–4 ug/mL^f^	remi	40–60	QoR-15	Korea
Lee 2024(a) [38]	54/50	64/53	NA	72	I-III	Spine surgery	118/118	1–2	3 ug/mL^f^	remi	25–50^e^	QoR-15	Korea
Lee 2024(b) [44]	54/54	0	24/24	63	I-II	Breast surgery	55/60	1–2	4 ug/mL^f^	remi	40–60	QoR-15	Korea
Luo 2023^a^ [39]	37–41	44–68	23–24	192	I-II	Laparoscopic surgery	43–46	1–2-3	6	remi	40–60	QoR-40	China
Mao 2022 [40]	53/50	64/70	25/24	128	I-III	Urologic surgery	79/63	1–2	4–10	remi	40–60	QoR-15	China
Tang 2023 [41]	49/50^d^	48/47	25/24	114	I-II	AMR	47/47	0.4–2	1–3 ug/mL^f^	remi	40–60	QoR-15	China
Zhao 2023 [42]	65/65	65/61	21/22	108	I-II	LRE	286/282	0.4–1	4–10	remi	45–60	QoR-15	China

(a) and (b) after the study name indicate different studies by distinct authors, used to differentiate between them

^a^Four-arm study

^b^Intraoperative infusion dose

^c^Data are presented as remimazolam/propofol groups

^d^Presented as median; *VCP* Vocal cord polypectomy, *LRE* Laparoscopic radical esophagectomy, *AMR* Arthroscopic meniscus repair, *OMS* oral and maxillofacial surgery

^e^Patient state index provided by SedLine® monitor; *BMI* body mass index, *BIS* Bispectral Index, *QoR* quality of recovery

^f^Target effect-site concentration; *remi* remifentanil, *TAVI* Transcatheter Aortic Valve Implantation, *ASA* American Society of Anesthesiologists Physical Status Classification, *NA* not available

### Quality of the studies

**Fig. 2:**
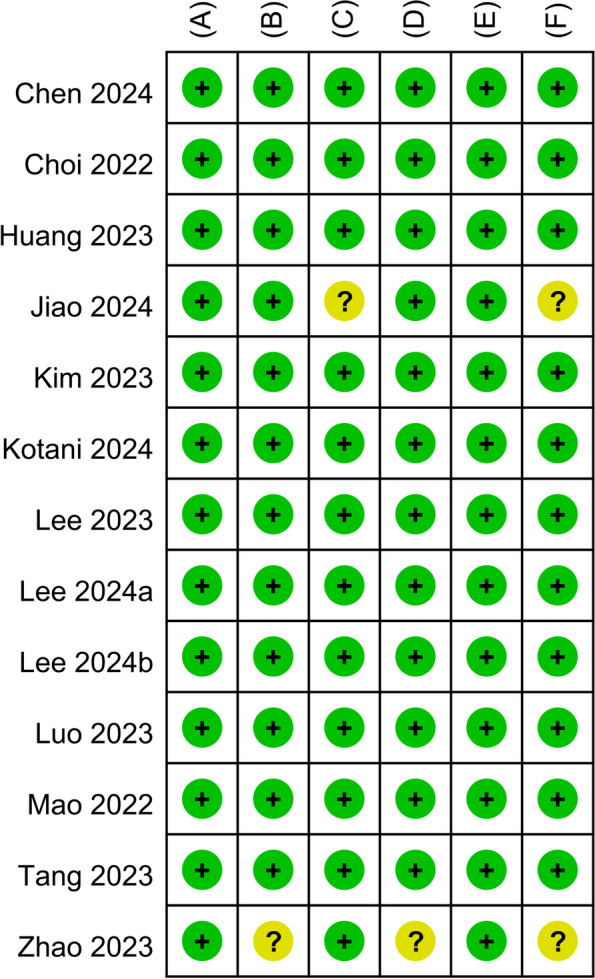
Risk of bias assessment across studies. **A** Bias arising from the randomization process. **B** Bias due to deviations from intended interventions. **C** Bias due to missing outcome data. **D** Bias in measurement of the outcome. **E** Bias in selection of the reported result. **F** Overall risk of bias

The risk-of-bias assessment led to the identification of potential concerns in some domains of the two studies included (Fig. 2). In the trial conducted by Jiao et al., 18 patients were excluded after randomization, which could have introduced bias. In the trial by Zhao et al., the lack of blinding of patients, study personnel, and outcome assessors could have influenced the administered interventions and introduced bias in the outcome evaluations, particularly for the subjective measures. In eight studies [33, 35–41], the anesthesiologists were not blinded to the anesthetics used. However, this lack of blinding is unlikely to have influenced the assessed outcomes. Consequently, the risk of bias stemming from deviations from the intended interventions was deemed low in these studies. In summary, the overall risk of bias was considered to be of some concern in 2 studies [34, 42] and low in the remaining 11 studies [32, 33, 35–41, 43, 44] (Fig. 2).

### Outcomes

#### Primary outcome

**Fig. 3:**
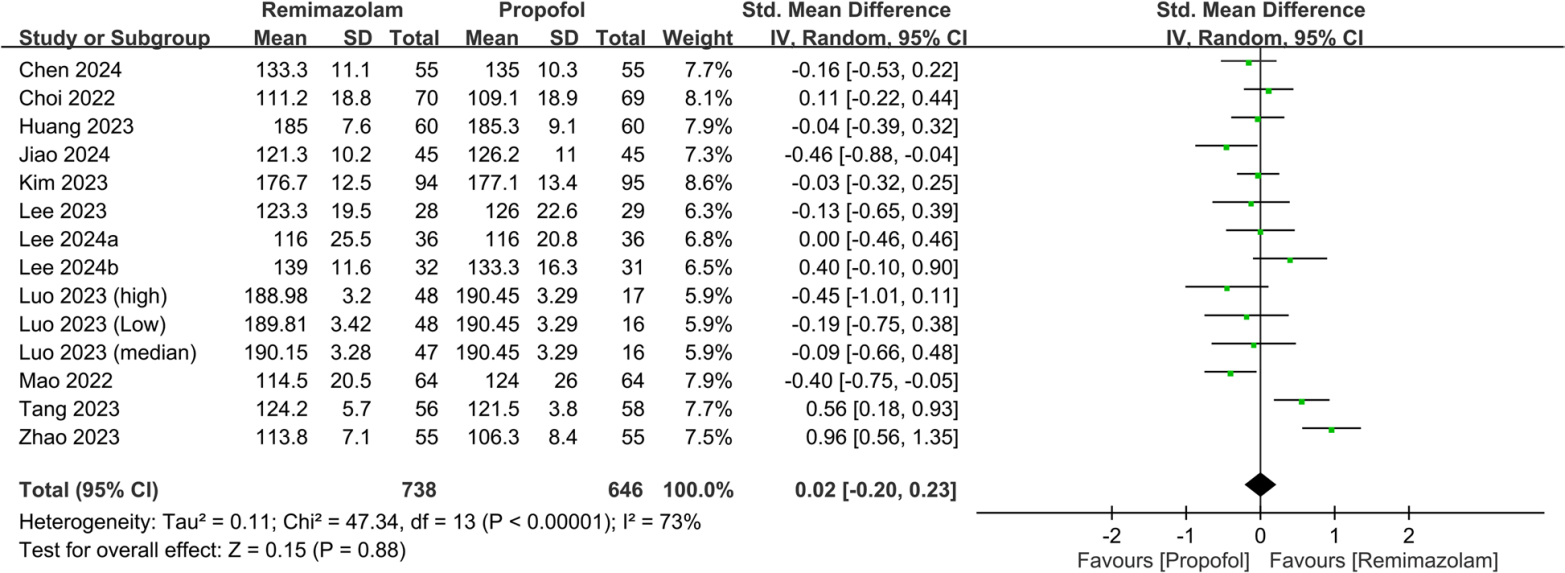
Forest plot showing quality of recovery (QoR) at postoperative day (POD)1. SD: standard deviation; IV: invariance; Std: standardized

The meta-analysis comparing the effects of remimazolam and propofol on postoperative QoR on POD 1 included 14 datasets from 12 studies, with a total of 1,384 participants (738 in the remimazolam group and 646 in the propofol group). The pooled results indicated a negligible difference in the mean recovery scores between remimazolam and propofol (SMD: 0.02, 95% CI: − 0.20 to 0.23, *P* = 0.88, *I*^*2*^ = 73%) (Fig. 3), suggesting that there is no significant advantage of one drug over the other in terms of QoR.

**Fig. 4:**
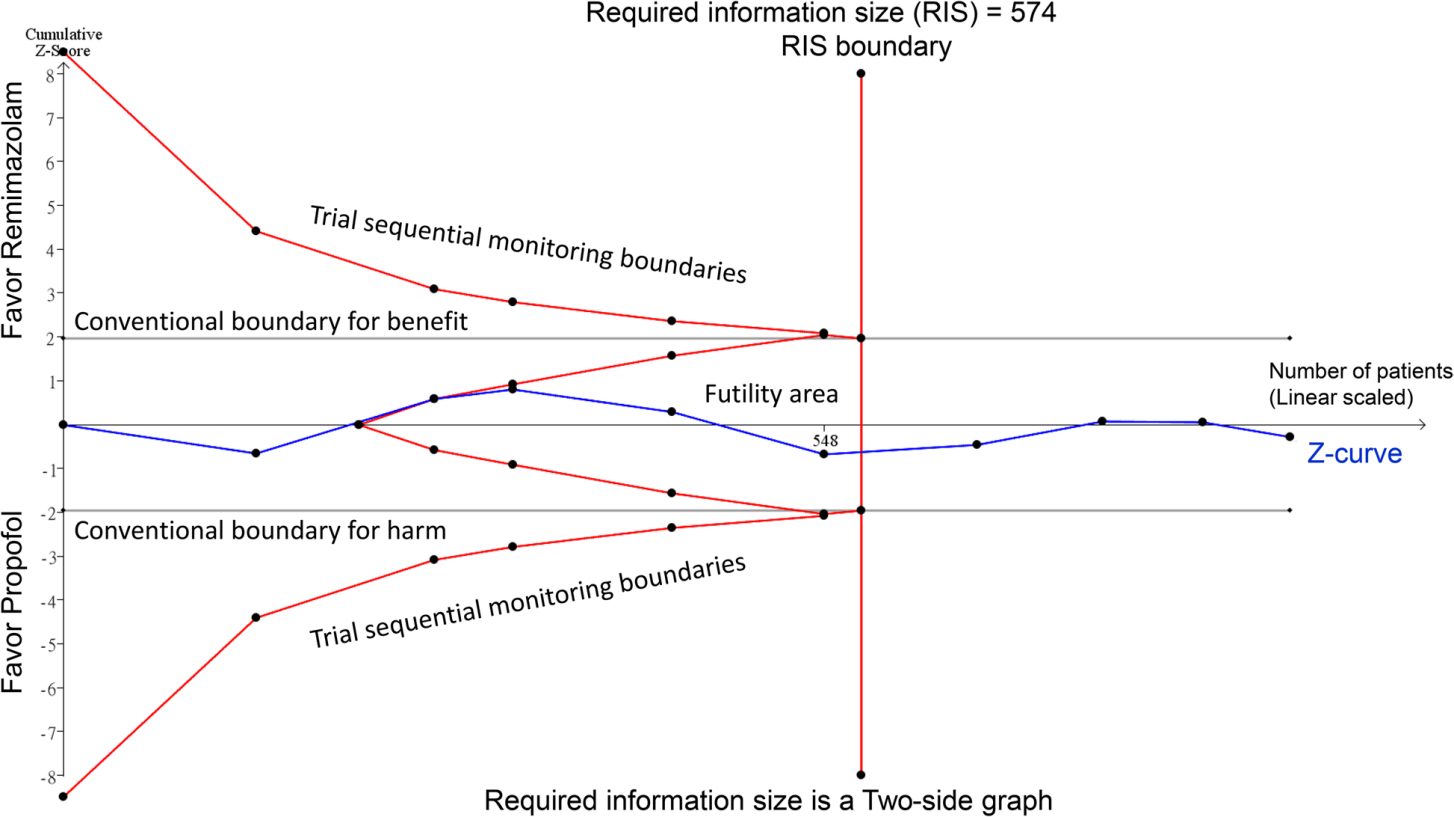
Trial sequential analysis (TSA) was conducted to evaluate the adequacy of the information size and the robustness of evidence concerning the QoR-15 scale. The TSA was configured with an alpha level of 0.05, a power of 80%, and a mean difference of 6.0, reflecting the minimal clinically important difference for the QoR-15 scale. The z-curve, representing the cumulative evidence, intersects the futility boundary and surpasses the required information size (RIS) boundary, indicating that sufficient evidence exists to support no significant difference between remimazolam and propofol on the QoR-15 scale

TSA was conducted with a focus on the QoR-15 scale to evaluate the strength of the evidence. As shown in Fig. 4, the z-curve intersected the futility boundary and reached the required information size, indicating that there is sufficient evidence to support the lack of difference in the QoR-15 scale outcomes between remimazolam and propofol.

#### Secondary outcome

**Fig. 5:**
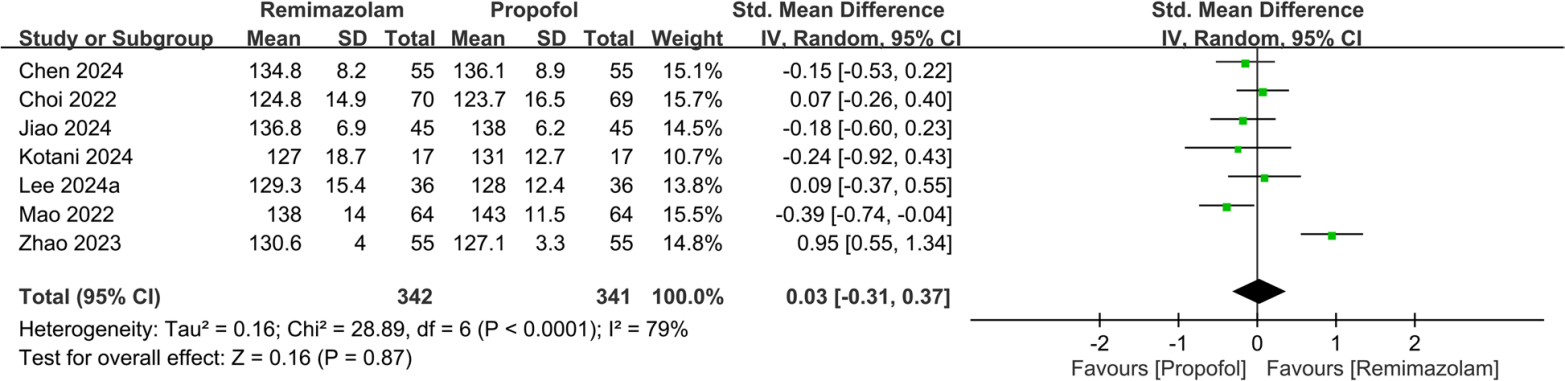
Forest plot showing no difference in quality of recovery (QoR) scores between remimazolam and propofol at postoperative day (POD) 2–3. SD: standard deviation; IV: invariance; Std: standardized

**Fig. 6:**
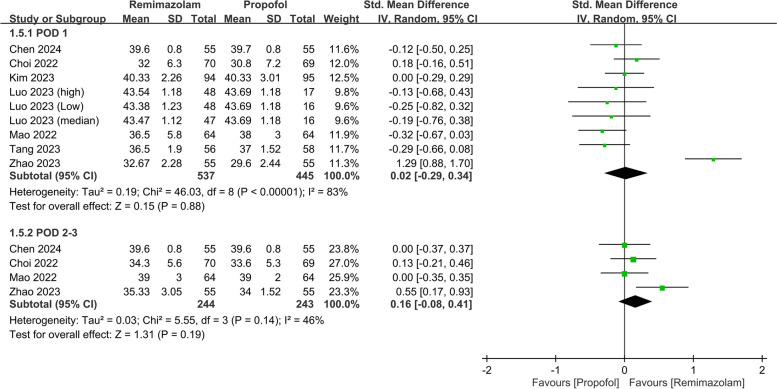
Forest plot showing no difference in emotional status dimension between remimazolam and propofol at postoperative day (POD) 1 and POD 2–3. SD: standard deviation; IV: invariance; Std: standardized

**Fig. 7:**
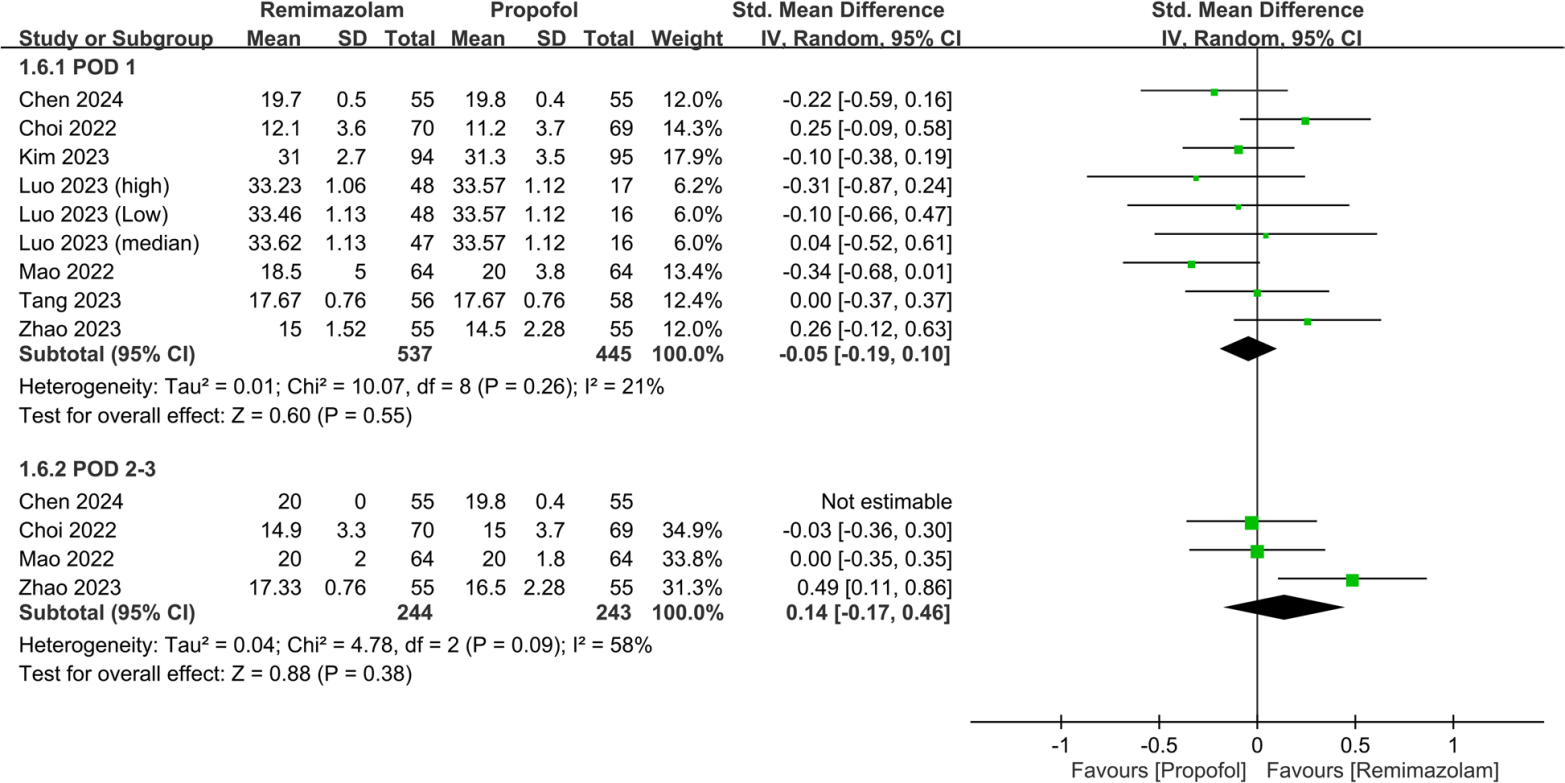
Forest plot showing no difference in pain dimension between remimazolam and propofol at postoperative day (POD) 1 and POD 2–3. SD: standard deviation; IV: invariance; Std: standardized

**Fig. 8:**
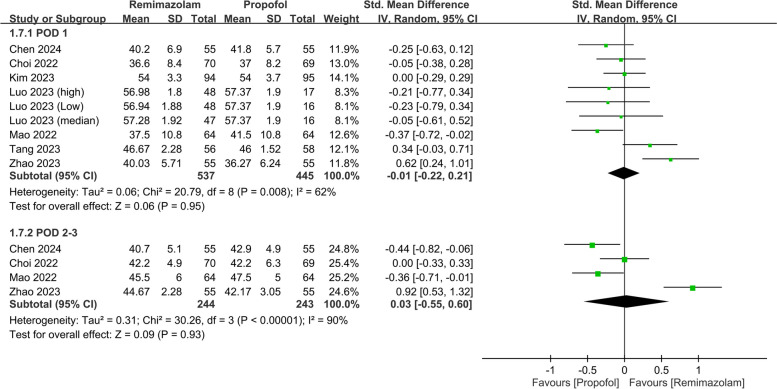
Forest plot showing no difference in physical comfort dimension between remimazolam and propofol at postoperative day (POD) 1 and POD 2–3. SD: standard deviation; IV: invariance; Std: standardized

**Fig. 9:**
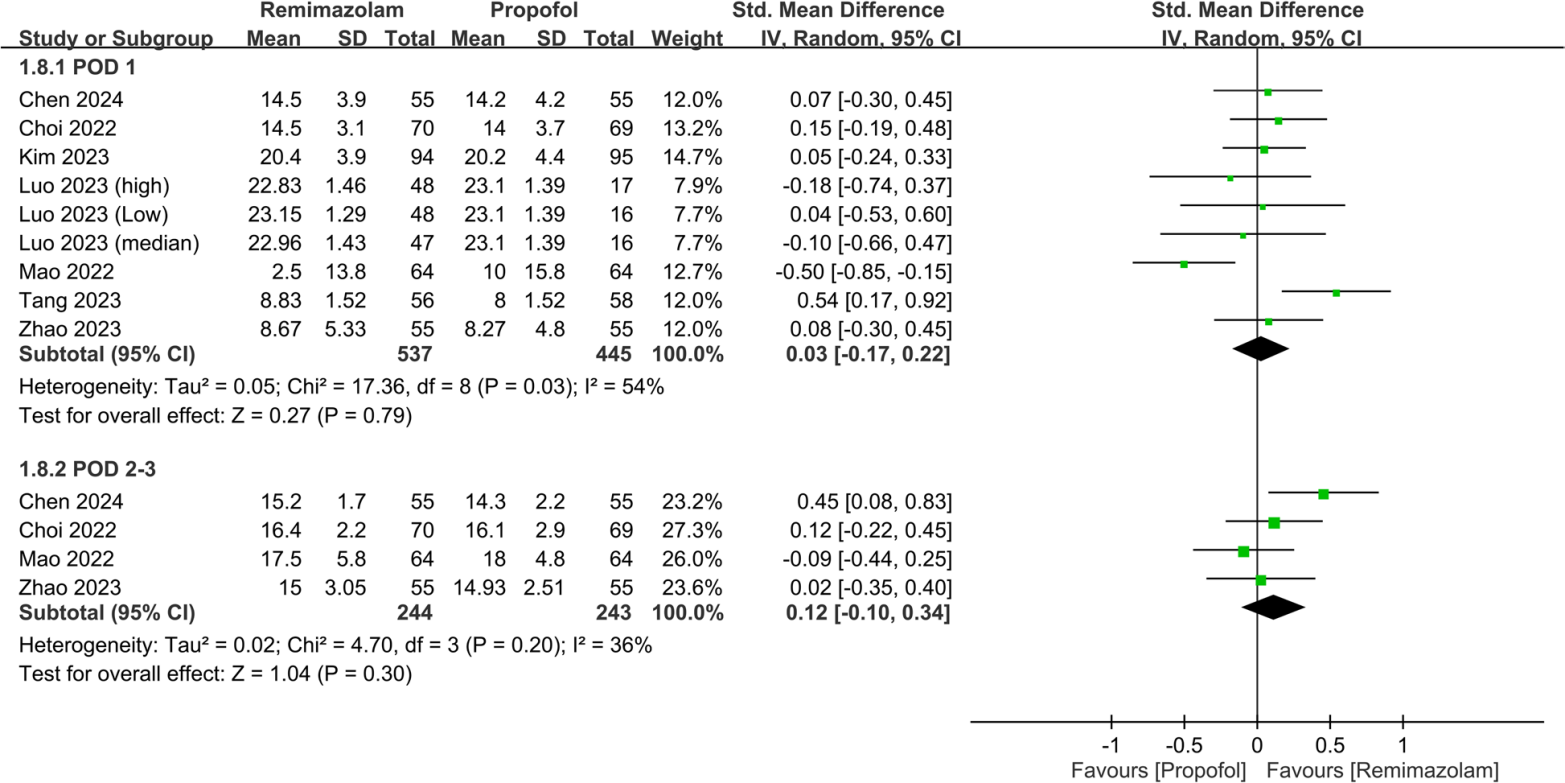
Forest plot showing no difference in physical independence dimension between remimazolam and propofol at postoperative day (POD) 1 and POD 2–3. SD: standard deviation; IV: invariance; Std: standardized

**Fig. 10:**
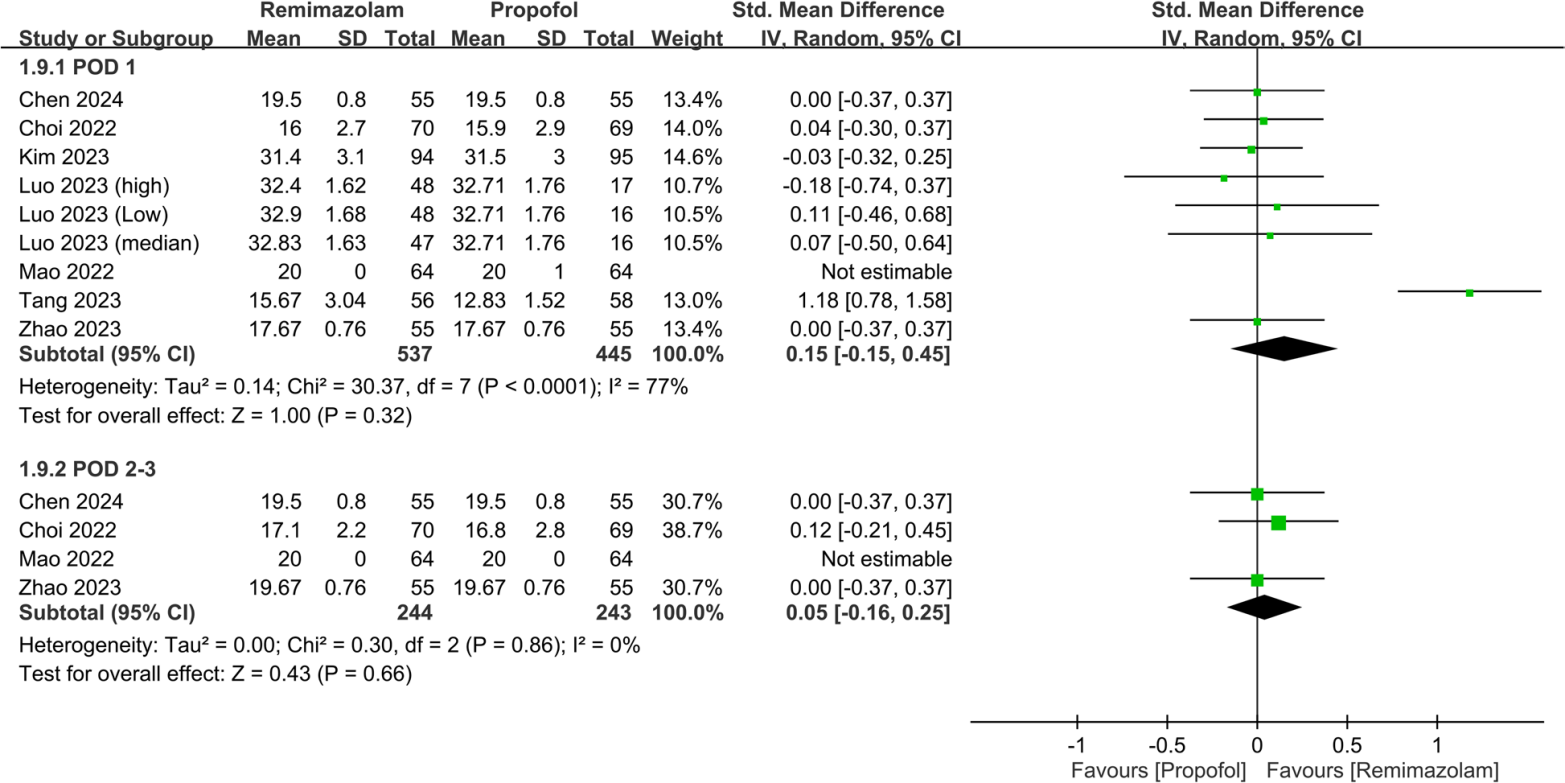
Forest plot showing no difference in psychological support dimension between remimazolam and propofol at postoperative day (POD) 1 and POD 2–3. SD: standard deviation; IV: invariance; Std: standardized

The pooled analysis also revealed a negligible difference in the QoR scores between the two anesthetics (SMD: 0.03, 95% CI: − 0.31, 0.37, *P* = 0.87, *I*^*2*^ = 79%) on PODs 2–3 (Fig. 5). Nine studies involving 982 patients evaluated various QoR dimensions on POD 1. The differences in these QoR dimensions between the groups were minimal, with the following metrics: emotional status (SMD: 0.02, 95% CI: − 0.29, 0.34, *P* = 0.88, *I*^*2*^ = 83%) (Fig. 6), pain (SMD: − 0.05, 95% CI: − 0.19, 0.10, *P* = 0.55, *I*^*2*^ = 21%) (Fig. 7), physical comfort (SMD: − 0.01, 95% CI: − 0.22, 0.21, *P* = 0.95, *I*^*2*^ = 62%) (Fig. 8), physical independence (SMD: 0.03, 95% CI: − 0.17, 0.22, *P* = 0.79, *I*^*2*^ = 54%) (Fig. 9), and psychological support (SMD: 0.15, 95% CI: − 0.15, 0.45, *P* = 0.32, *I*^*2*^ = 77%) (Fig. 10). These results indicated that there were no significant differences in these QoR dimensions on POD 1 between patients administered remimazolam and those administered propofol. Similarly, on PODs 2–3, no differences were observed in these QoR dimensions between the groups.

The aggregated data indicated that patients administered propofol lost consciousness significantly faster than those administered remimazolam (MD: 32.27 s, 95% CI: 16.52, 48.02, *P* < 0.001, *I*^*2*^ = 97%) (Supplemental Fig. 1). However, patients administered remimazolam regained consciousness more quickly than those administered propofol (MD: − 1.60 min, 95% CI: − 2.90, − 0.31, *P* = 0.02, *I*^*2*^ = 96%) (Supplemental Fig. 2). However, no significant difference was observed in time to extubation between the two anesthetic agents (MD: − 0.87 min, 95% CI: − 2.76, 1.03, *P* = 0.37, *I*^*2*^ = 97%) (Supplemental Fig. 3). The incidence of emergence agitation in patients administered remimazolam versus propofol during general anesthesia was similar (RR: 0.81, 95% CI: 0.44, 1.48, *P* = 0.49, *I*^*2*^ = 0%) (Supplemental Fig. 4). Furthermore, there was no difference in the length of PACU stay between the groups (MD: 0.28 min, 95% CI: − 0.43, 0.98, *P* = 0.44,* I*^*2*^ = 0%) (Supplemental Fig. 5). The analysis based on data from 12 studies revealed that the risk of PONV between the groups was also not statistically significant (RR: 1.12, 95% CI: 0.71, 1.76, *P* = 0.62, *I*^*2*^ = 0%) (Supplemental Fig. 6). Interestingly, patients administered remimazolam exhibited a lower risk of rescue analgesia requirement in the PACU than those administered propofol (RR = 0.62, 95% CI: 0.43–0.89, *P* = 0.009, *I*^*2*^ = 0%) (Supplemental Fig. 7). However, this difference was not evident in the ward (RR = 1.39, 95% CI: 0.89–2.16, *P* = 0.14, *I*^*2*^ = 0%) (Supplemental Fig. 7).

#### Sensitivity analysis

The sensitivity analyses conducted using the leave-one-out approach for all outcomes are detailed in Table 2. These analyses confirmed that the majority of the results were robust and independent of any single study (Table 2). However, two outcomes—time to ROC and rescue analgesia requirement in the PACU—were exceptions, indicating that the findings might have been influenced by specific studies. Additionally, we planned to conduct a sensitivity analysis by removing studies deemed to have a high risk of bias. However, since none of the studies included in our meta-analysis were deemed to have a high risk of bias, we chose not to perform this specific sensitivity analysis.

**Table 2:**
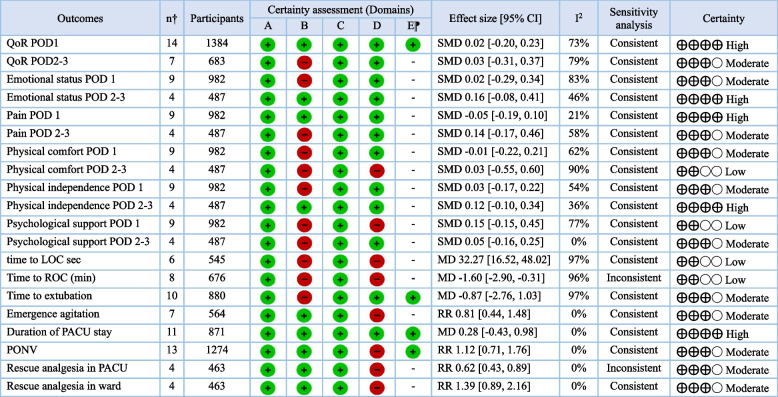
Summary of outcomes and certainty of evidence based on the Grading of Recommendations Assessment, Development and Evaluation (GRADE) approach

A: risk of bias; B: Inconsistency; C: Indirectness; D: Imprecision; E: publication bias;  Green circular icon: not serious; Red circular icon: serious

*RR* Risk Ratio, *SMD* Standardized Mean Difference, *CI* Confidence Interval, *POD* postoperative day, *LOC* Loss of Consciousness,  *ROC* Return of Consciousness, *PACU* Post-Anesthesia Care Unit, *PONV* Postoperative Nausea and Vomiting, ⁋publication bias assessed when more than ten studies or datasets were available for a given outcome; †number of studies or datasets

#### Publication bias

The potential for publication bias was evaluated for four outcomes, each supported by more than 10 studies, using funnel plots. The plots indicated low risks of publication bias for the following outcomes: QoR on POD 1 (Supplemental Fig. 8), time to extubation (Supplemental Fig. 9), length of PACU stay (Supplemental Fig. 10), and risk of PONV (Supplemental Fig. 11), as evidenced by the symmetry in the funnel plots.

#### Meta-regression analysis for primary outcome

**Fig. 11:**
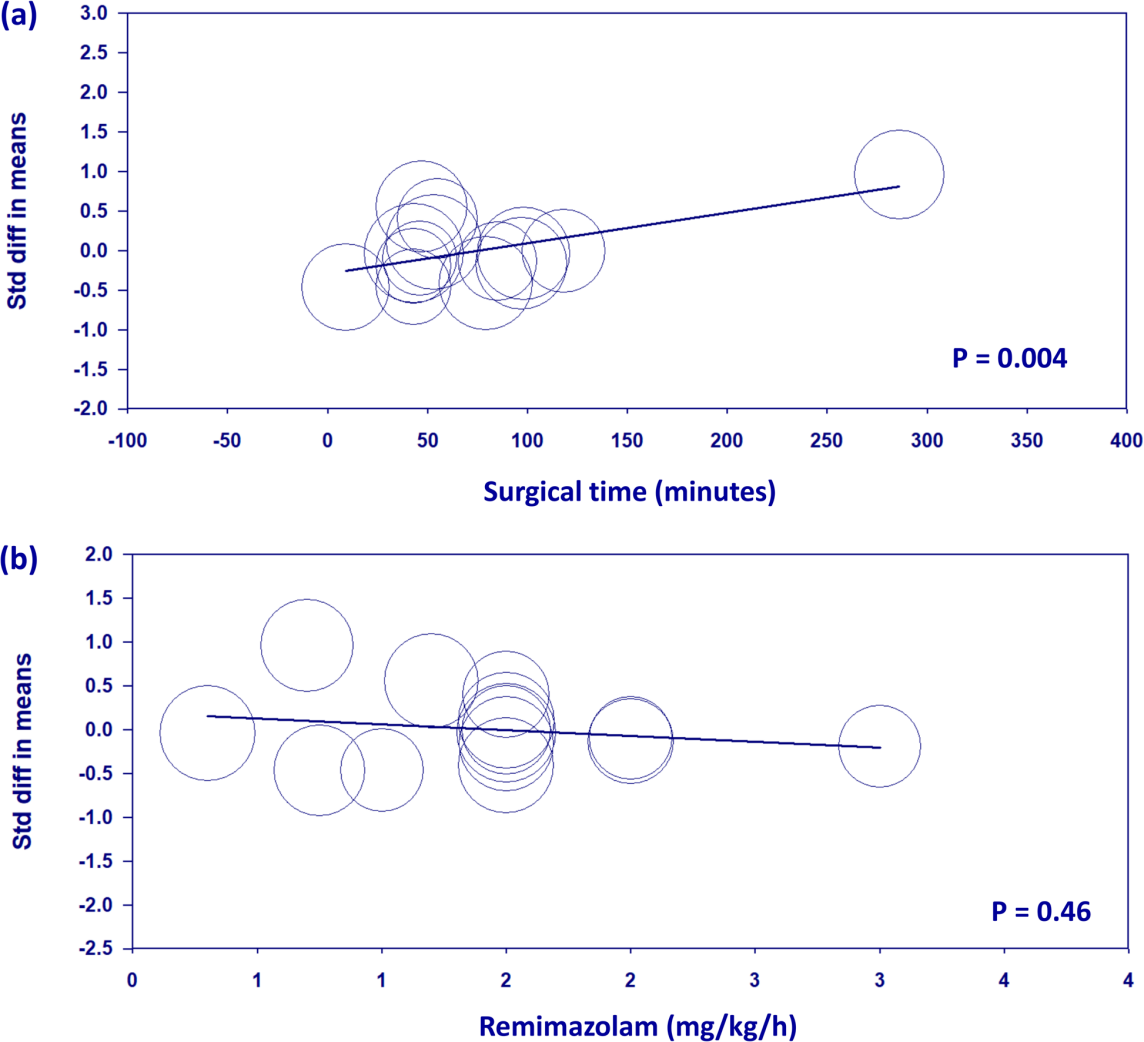
Meta-regression analyses exploring the influence of (**a**) surgical time and (**b**) remimazolam dose on the standardized mean difference (SMD) in quality of recovery (QoR) scores between remimazolam and propofol on postoperative day 1. The size of each circle represents the weight of the study in the meta-regression. The solid line represents the weighted regression line. A significant association was observed between surgical time and the SMD in QoR scores (*P* = 0.004), with longer surgical times favoring remimazolam. No significant association was found between remimazolam dose and the SMD in QoR scores (*P* = 0.46). Std diff: standardized difference

Meta-regression analysis revealed a significant association between the duration of surgery and SMD in QoR scores between remimazolam and propofol (*P* = 0.004) (Fig. 11a). This finding suggests that as the duration of surgery increases, the difference in QoR scores between the two anesthetic agents becomes more favorable for remimazolam. In other words, the advantage of remimazolam over propofol in terms of postoperative QoR increases with longer surgical procedures. In contrast, the meta-regression analysis for remimazolam dose did not show a significant association with SMD in QoR scores between the two anesthetic agents (*P* = 0.46) (Fig. 11b). This finding indicates that the dose of remimazolam used for anesthesia maintenance did not significantly influence the difference in postoperative QoR between remimazolam and propofol.

#### Certainty of evidence

The summary of findings and certainty of evidence are shown in Table 2. The certainty of evidence for the primary outcome was high. For other secondary outcomes, the certainty of evidence for majority of the outcomes was moderate to high. Only four outcomes were considered to have low certainty of evidence, namely, physical comfort on PODs 2–3, psychological support on POD 1, time to LOC, and time to ROC.

## Discussion

This meta-analysis compared the effects of remimazolam and propofol on postoperative QoR in surgical patients who underwent general anesthesia. No significant differences were found in QoR scores, QoR dimensions, emergence agitation, length of PACU stay, or PONV risk. However, remimazolam was associated with slower anesthetic induction, faster ROC, and lower risk of rescue analgesia requirement in the PACU. The certainty of evidence was high for the primary outcome and moderate to high for most secondary outcomes. These findings suggest that remimazolam is a potential alternative to propofol for general anesthesia, offering similar QoR with advantages in consciousness recovery and immediate postoperative analgesia.

Our meta-analysis specifically focused on comparing the QoR of remimazolam and propofol for general anesthesia. A previous meta-analysis by Peng et al. included QoR as a secondary outcome, but only analyzed global QoR-15 scores on postoperative day 1 without looking at the individual QoR dimensions [24]. Although Peng et al. assessed the robustness of their evidence using TSA, they concluded that it was insufficient [24]. On the other hand, another meta-analysis by Qin et al. [27] explored individual dimensions of QoR but did not incorporate TSA to evaluate the strength of the evidence. Our meta-analysis provides a more detailed analysis of QoR by examining QoR scores on both POD 1 and 2–3, as well as looking at individual QoR dimensions. This allowed for a more comprehensive comparison of the recovery profiles of the two drugs. Furthermore, our meta-analysis included a larger number of studies (13 RCTs) and 1418 patients compared to previous meta-analyses [24, 27] that only included two RCTs that focused on QoR. Our larger sample size increased the statistical power and precision of our pooled effect estimates. Our TSA also validated the adequacy of the sample size and the robustness of the evidence. This strengthens the reliability of our finding that there was no significant difference in QoR between remimazolam and propofol.

The implications of these findings are significant for clinical practice. First, the results indicated that remimazolam can be considered as a viable alternative to propofol for general anesthesia as it provides similar postoperative QoR to the latter across various domains. This provides anesthesiologists with more options for tailoring anesthetic management to individual patient needs and preferences. Remimazolam has been associated with a lower risk of hypotension than propofol [21, 45, 46]. Consequently, the use of remimazolam may provide comparable QoR without carrying the risk of hypotension, which is particularly important for patients with cardiovascular comorbidities or those undergoing procedures with a high risk of hemodynamic instability. Second, the comparable recovery profiles of remimazolam and propofol may have economic implications, as the choice between these two could be based on factors such as cost, availability, and institutional protocols without compromising patient outcomes. Finally, in elderly patients who underwent sedative gastrointestinal endoscopy, remimazolam significantly lowered the risk of respiratory depression, hypoxemia, and hypotension compared with propofol [46]. In ambulatory surgery or sedation settings where rapid recovery is crucial, the comparable QoR of both agents, coupled with the pulmonary hemodynamic stability of remimazolam, supports its use.

Our meta-regression analyses provided additional insights into the potential influence of surgical duration and remimazolam dose on the comparison between remimazolam and propofol in terms of postoperative QoR. The significant association between surgical duration and difference in QoR scores suggests that the advantage of remimazolam over propofol increases with longer surgical procedures. This finding may have clinical implications for anesthesiologists when selecting an appropriate anesthetic agent based on the anticipated duration of surgery. However, due to the fact that only one study included addressed prolonged surgery, additional research is necessary to confirm our findings. The lack of a significant association between remimazolam dose and the difference in QoR scores suggests that the dose of remimazolam used for anesthesia maintenance may not be a critical factor in determining the postoperative QoR compared to propofol. However, it is important to acknowledge that the range of remimazolam doses used in the included studies may not have been wide enough to detect a significant dose–response relationship.

Patients administered propofol had a significantly faster time to LOC than those administered remimazolam (MD: 32.27 s). This finding suggests that propofol in the dosage studied is more suitable for rapid anesthetic induction, which could be advantageous in certain clinical situations, such as emergency surgeries or surgeries involving patients with a high risk of aspiration [47, 48]. Conversely, patients administered remimazolam had a significantly faster time to ROC than those administered propofol (MD: − 1.60 min). However, it is noteworthy that the clinical significance of this difference may be relatively small. Prolonged time to extubation, longer PACU stay, and emergence agitation can increase healthcare costs and reduce operating room efficiency [49, 50]. Therefore, both anesthetic agents may be cost effective in terms of operating room utilization. PONV is a common and distressing side effect of general anesthesia that can adversely affect patient recovery and satisfaction [51]. Propofol is recognized for its beneficial effect in PONV risk reduction compared with inhalation-based anesthesia [16]. The comparable risk of PONV between the two anesthetic agents in the current meta-analysis suggests that remimazolam could be an alternative to propofol, particularly for patients concerned about the likelihood of experiencing PONV. Our findings regarding the risk of PONV agree with those of previous meta-analyses, which also demonstrated a similar risk of PONV between remimazolam and propofol in sedation settings [46].

An interesting finding from our current meta-analysis is that the need for rescue analgesia in the PACU is lower with remimazolam than with propofol. Remifentanil-induced hyperalgesia [52, 53], which can increase pain intensity after surgery, exerts a significant effect 1 h postoperatively and minimal effect 24 h postoperatively [32]. Prophylactic agents such as N-methyl-D-aspartate receptor antagonists and dexmedetomidine have proven effective in reducing remifentanil-induced hyperalgesia [52, 54, 55]. Our findings suggest that remimazolam helps mitigate this effect of remifentanil-induced hyperalgesia. The shorter time to ROC with remimazolam than with propofol suggests that this beneficial effect is likely not due to residual sedative properties. The potential mechanisms, which may involve interactions with GABA receptors and impacts on neural processing at both the spinal and supraspinal levels [56–58], remain unclear. Nevertheless, due to the limited number of studies on this outcome, further research is warranted to corroborate our findings.

This meta-analysis has several limitations that need to be acknowledged. First, the included studies were mainly conducted on Asian populations, which may limit the generalizability of the findings to other ethnic groups. Second, the surgical procedures and anesthetic protocols varied among the studies, potentially contributing to heterogeneity in some outcomes. Third, majority of the included studies focused on short-term surgical procedures, and the impact of remimazolam on postoperative recovery in longer or more complex surgeries remain unclear. Fourth, the included studies did not provide sufficient data to assess the cost-effectiveness of remimazolam compared with propofol, which is an important consideration for the choice of anesthetic in clinical practice. Fifth, while the risk of bias was generally low in the included studies, some concerns were identified in a few domains, such as blinding of anesthesiologists and outcome assessors, which may have introduced biases in the reported outcomes. Sixth, we did not involve a subject librarian in developing our search strategy, which may have resulted in the omission of some relevant studies and potentially impacted the completeness of our meta-analysis. For future meta-analyses, involving a subject librarian is recommended to ensure a more thorough and reliable search process. Lastly, because only a few studies focused on elderly patients (i.e., aged > 65 years), the applicability of the results to this patient population may be limited. Thus, further research targeting this age group is warranted.

In conclusion, this meta-analysis provides high certainty evidence that remimazolam and propofol offer comparable postoperative QoR in surgical patients who underwent general anesthesia. The advantage of remimazolam over propofol appears to increase with longer surgical procedures, highlighting the importance of considering the anticipated duration of surgery when selecting between these two anesthetic agents. Despite some differences in induction and emergence times, the overall recovery profiles were similar between the two anesthetic agents. The potential advantage of remimazolam in reducing immediate postoperative analgesia requirement warrants further investigation. Future studies should focus on specific surgical populations, evaluate long-term outcomes, and compare the cost-effectiveness of remimazolam and propofol to facilitate decision-making in anesthesia management.

## Supplementary Information

Supplementary Material 1.

## Data Availability

The datasets used and/or analyzed in the current study are available from the corresponding author upon reasonable request.
