# Supplementary material for: Comparing subjective quality of recovery between remimazolam- and propofol-based total intravenous anesthesia for surgical procedures: a meta-analysis
Source: Syst Rev. 2024 Sep 17;13:235. doi: 10.1186/s13643-024-02660-8 (PMC11409698; doi:10.1186/s13643-024-02660-8)

**Supplemental table 1.** Search strategies for databases

| **Medline** | | |
| --- | --- | --- |
| 1 | ("(operative or surgical) adj4 (procedure* or technique*)" or "surger*" or "operation*").mp. |  |
| 2 | exp "Surgical Procedures, Operative"/ |  |
| 3 | ("Remimazolam" or "ONO 2745" or "CNS 7056" or "methyl 3-(8-bromo-1-methyl-6-(2-pyridinyl)-4H-imidazo(1,2-a)(1,4)benzodiazepin-4-yl)propanoate").mp. |  |
| 4 | ("Propofol " or "Disoprofol" or "2,6-Bis(1-methylethyl)phenol" or "2,6-Diisopropylphenol" or "2,6 Diisopropylphenol" or "Diprivan" or "Disoprivan" or "ICI-35,868" or "Aquafol" or "Fresofol" or "Ivofol" or "Recofol").mp. |  |
| 5 | ("quality of recovery score" or "QoR-40" or "Quality of Recovery-40" or "Quality of Recovery-15" or "QoR-15" or "Quality of Recovery scale" or "quality of recovery").mp. |  |
| 6 | (1 or 2) and 3 and 4 and 5 |  |
| 7 | 6 and (((randomized controlled trial or controlled clinical trial).pt. or randomi*ed.ab. or placebo.ab. or drug therapy.fs. or randomly.ab. or trial.ab. or groups.ab.) not (exp animals/ not humans.sh.)) |  |
| **Embase** | | |
| 1 | ("(operative or surgical) adj4 (procedure* or technique*)" or "surger*" or "operation*"):ti,ab,kw,de |  |
| 2 | "Surgical Procedures, Operative"/exp |  |
| 3 | ("Remimazolam" or "ONO 2745" or "CNS 7056" or "methyl 3-(8-bromo-1-methyl-6-(2-pyridinyl)-4H-imidazo(1,2-a)(1,4)benzodiazepin-4-yl)propanoate"):ti,ab,kw,de |  |
| 4 | ("Propofol " or "Disoprofol" or "2,6-Bis(1-methylethyl)phenol" or "2,6-Diisopropylphenol" or "2,6 Diisopropylphenol" or "Diprivan" or "Disoprivan" or "ICI-35,868" or "Aquafol" or "Fresofol" or "Ivofol" or "Recofol"):ti,ab,kw,de |  |
| 5 | ("quality of recovery score" or "QoR-40" or "Quality of Recovery-40" or "Quality of Recovery-15" or "QoR-15" or "Quality of Recovery scale" or "quality of recovery"):ti,ab,kw,de |  |
| 6 | (1 or 2) and 3 and 4 and 5 |  |
| 7 | 6 and (((randomized controlled trial or controlled clinical trial).pt. or randomi*ed.ab. or placebo.ab. or drug therapy.fs. or randomly.ab. or trial.ab. or groups.ab.) not (exp animals/ not humans.sh.)) |  |
| **Cochrane library** | |  |
| #1 | ("(operative or surgical) adj4 (procedure* or technique*)" or "surger*" or "operation*"):ti,ab,kw |  |
| #2 | [mh "Surgical Procedures, Operative"] |  |
| #3 | ("Remimazolam" or "ONO 2745" or "CNS 7056" or "methyl 3-(8-bromo-1-methyl-6-(2-pyridinyl)-4H-imidazo(1,2-a)(1,4)benzodiazepin-4-yl)propanoate"):ti,ab,kw |  |
| #4 | ("Propofol " or "Disoprofol" or "2,6-Bis(1-methylethyl)phenol" or "2,6-Diisopropylphenol" or "2,6 Diisopropylphenol" or "Diprivan" or "Disoprivan" or "ICI-35,868" or "Aquafol" or "Fresofol" or "Ivofol" or "Recofol"):ti,ab,kw |  |
| #5 | ("quality of recovery score" or "QoR-40" or "Quality of Recovery-40" or "Quality of Recovery-15" or "QoR-15" or "Quality of Recovery scale" or "quality of recovery"):ti,ab,kw |  |
| #6 | (#1 or #2) and #3 and #4 and #5 |  |
| **Google Scholar** | | |
| A hand-search strategy was used to identify the related studies, followed by using a forward snowballing strategy to retrieve all potentially eligible articles. | | |

**Supplemental figure 1**. Forest plot showing difference in loss of consciousness (LOC) between remimazolam and propofol


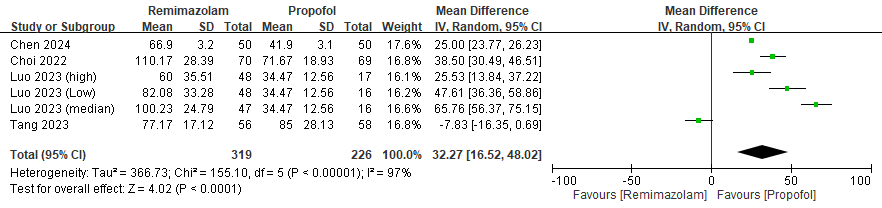


**Supplemental figure 2**. Forest plot showing difference in return of consciousness (ROC) between remimazolam and propofol


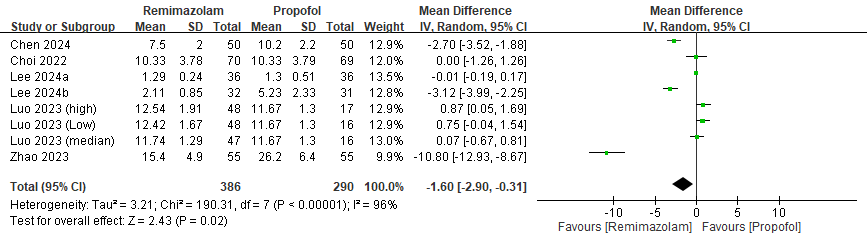


**Supplemental figure 3.** Forest plot showing difference in time to extubation between remimazolam and propofol


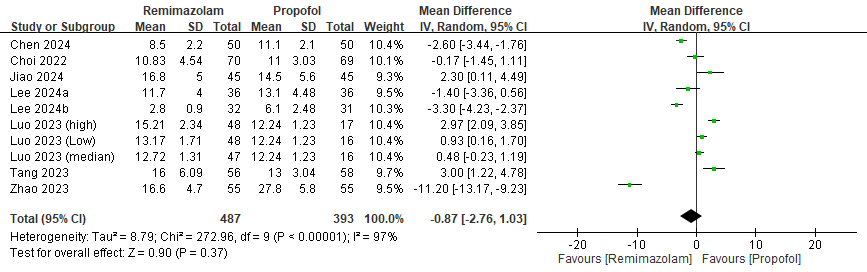


**Supplemental figure 4.** Forest plot showing incidence in emergence agitation between remimazolam and propofol


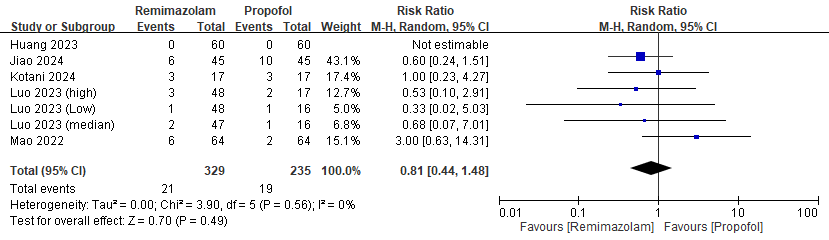


**Supplemental figure 5.** Forest plot showing difference in PACU stay between remimazolam and propofol


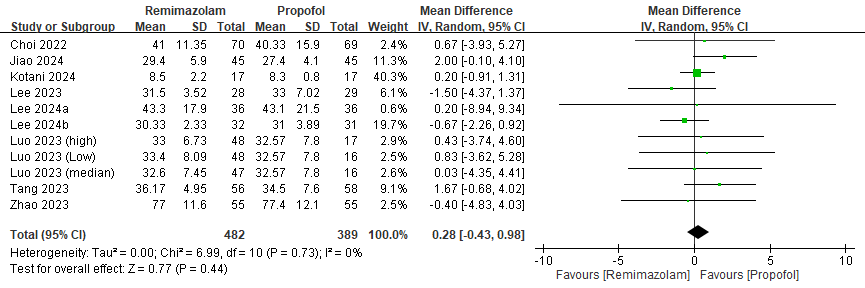


**Supplemental figure 6.** Forest plot showing incidence in postoperative nausea and vomiting (PONV) between remimazolam and propofol


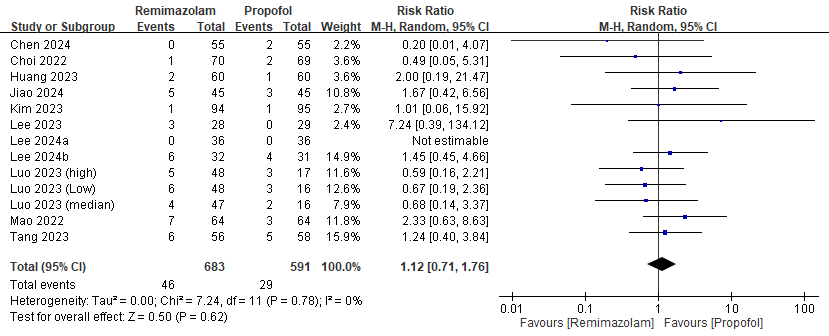


**Supplemental figure 7.** Forest plot showing incidence in rescue analgesia in the Post-Anesthesia Care Unit (PACU) or ward between remimazolam and propofol


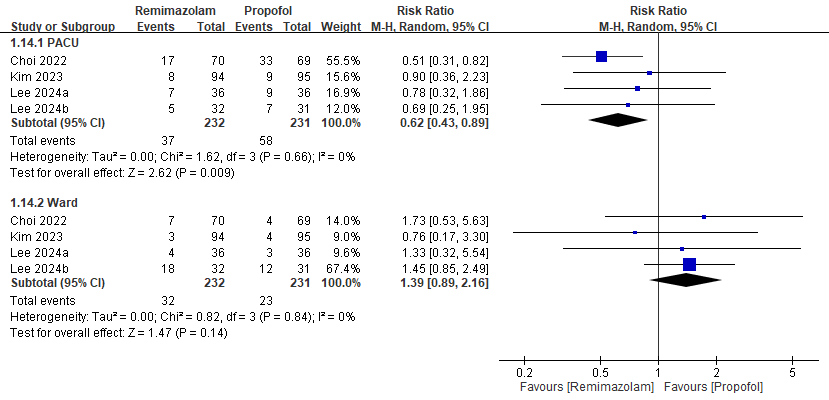


**Supplemental figure 8.** Assessing publication bias in studies on the quality of recovery (QoR) at postoperative day (POD) 1


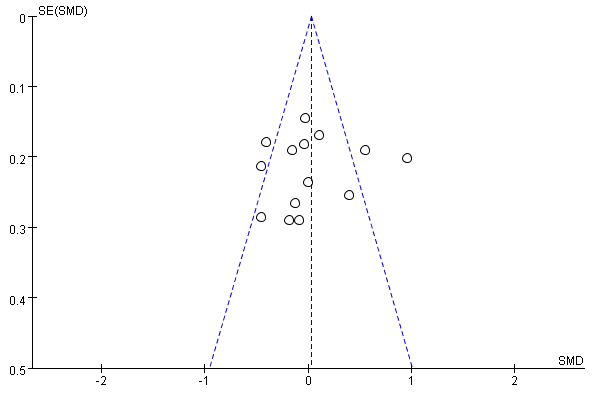


**Supplemental figure 9.** Assessing publication bias in studies on time to extubation


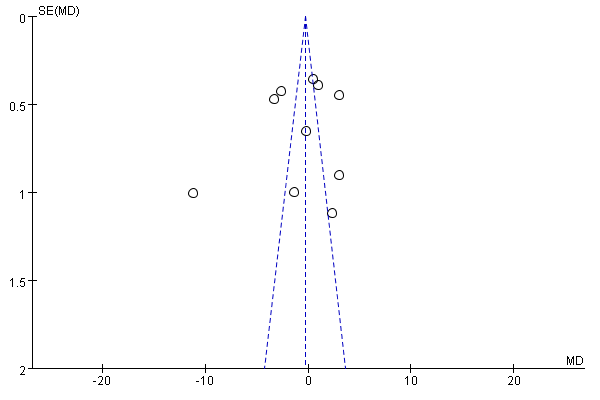


**Supplemental figure 10.** Assessing publication bias in studies on the duration of stay in the PACU


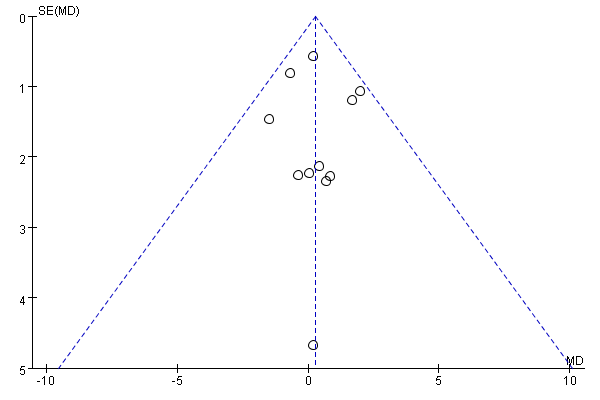


**Supplemental figure 11.** Assessing publication bias in studies on postoperative nausea and vomiting


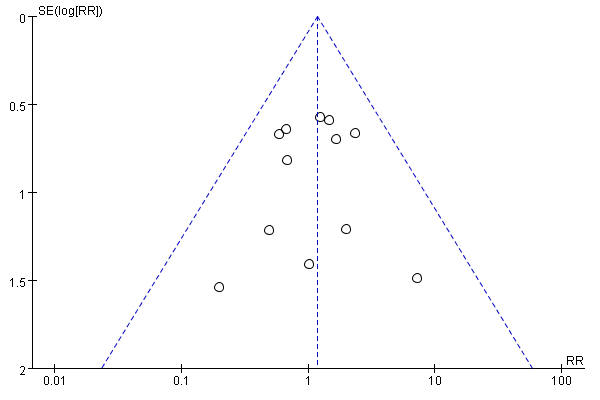

Supplement: Supplementary file 1 — Supplementary Material 1. [file 13643_2024_2660_MOESM1_ESM.docx]
